# Adverse events of immune checkpoint inhibitors for patients with digestive system cancers: A systematic review and meta-analysis

**DOI:** 10.3389/fimmu.2022.1013186

**Published:** 2022-10-21

**Authors:** Liqiu Kou, Qinglian Wen, Xiaolu Xie, Xiu Chen, Jun Li, Yaling Li

**Affiliations:** ^1^ Department of Pharmacy, The Affiliated Hospital of Southwest Medical University, Luzhou, China; ^2^ School of Pharmacy, Southwest Medical University, Luzhou, China; ^3^ Department of Oncology, The Affiliated Hospital of Southwest Medical University, Luzhou, China; ^4^ Department of Traditional Chinese Medicine, The Affiliated Hospital of Southwest Medical University, Luzhou, China

**Keywords:** immune checkpoint inhibitors, treatment-related adverse events, immune-related adverse events, digestive system cancers, systematic evaluation, advance intervention

## Abstract

**Objective:**

To study the incidence and distribution of adverse events in immune checkpoint inhibitors (ICI) for digestive system cancers and to provide a reference for the safe, rational, and effective use of immune detection site inhibitors.

**Methods:**

We searched for articles published in English between January 1, 2010, and May 18, 2022. All clinical trials of ICI-based therapies for digestive system cancers were investigated, including only randomized controlled trials that reported data on the overall incidence of treatment-related adverse events (trAEs) or immune-related adverse reactions (irAEs) or tables.

**Results:**

We searched 2048 records, of which 21 studies (7108 patients) were eligible for inclusion. The incidence of ICI trAEs of any grade was 82.7% (95% CI 73.9-90.0), and the incidence of grade 3 or higher trAEs was 27.5% (95% CI 21.3-34.1). The pooled rate of ICI irAEs of any grade was 26.3% (95% CI 11.8-44.0), and the incidence of grade 3 or higher irAEs was 9.4% (95% CI 1.1-24.6). In multivariate analysis, the incidence, characteristics, and distribution of AEs varied by cancer type, combination therapy modality (single/two-drug), and different agent types.

**Conclusion:**

Our meta-analysis summarizes AEs associated with ICI in digestive system cancers. The incidence, characteristics, and distribution of AEs vary by cancer type, combination therapy modality, and different agent types. These findings can be considered for the early identification of AEs and provide effective interventions to reduce the severity of these patients. It can provide a clinical reference and may contribute to clinical practice.

## Introduction

Immune checkpoint inhibitors (ICIs) works by blocking tumor cells [programmed cell death 1 Ligand-1 (PD-L1)] or T lymphocytes [programmed cell death protein-1 (PD-1) or cytotoxic T lymphocyte-associated protein 4 (CTLA-4)], resulting in an effective anti-tumor response in patients (1). In 2011, the CTLA-4 inhibitor Ipilimumab was approved for marketing by the FDA, becoming the world’s first approved immune checkpoint inhibitor drug (2). The discovery and clinical implementation of ICIs have revolutionized cancer treatment, bringing a new era of cancer therapy and improving the prognosis of patients with a variety of advanced cancers with this groundbreaking new approach (3). Currently, the FDA-approved immune checkpoint inhibitors are anti-CTLA-4 (Ipilimumab, tremelimumab, etc.), anti-PD-1 (pembrolizumab, toripalimab, nivolumab, etc.), and anti-PD-L (atezolizumab, durvaluma, etc.) (4).

ICIs have shown exciting clinical results in many tumor types (5, 6), but the practical application process is full of adverse effects, mainly including irAEs and trAEs. The mechanism of action of ICIs involves nonspecific activation of the immune system, and therefore, disruption of the critical role of checkpoint molecules in immune homeostasis may lead to autoimmune complications (7). IrAEs affect almost every organ in the body, most commonly the skin, gastrointestinal tract, lungs, endocrine, musculoskeletal and other systems (8). TrAEs are therapeutically relevant and appear mainly after treatment of malignancies with immune checkpoint inhibitors and encompass a larger spectrum than irAEs. TrAEs may cause by immune checkpoint inhibitors or other concomitant reactions (9). TrAEs may manifest in the hematological system, the skin, the Respiratory system, the urinary system, systemic reactions, etc (10). Some fatal toxicities can occur in 0.4%-1.2% of patients, such as myocarditis, meningitis, myasthenia gravis, and various neuropathies. Although relatively rare, they often exhibit significant diagnostic complexity and may be underestimated (11, 12). Given the potential for long-term survival, ICI-related adverse events become a particularly relevant consideration (13). For patients receiving ICI for cancer, most AEs are reversible if diagnosed and treated promptly (14). Therefore, understanding AEs are critical to ensure timely diagnosis and effective management of these potentially severe adverse events (15).

With the increasing clinical use of ICIs, there have been numerous reports of adverse events associated with ICIs (16–18). As the indications for ICIs continue to expand, ICIs have been widely used in gastric, hepatic, esophageal, and gastroesophageal junction cancers (19–21). But the AEs associated with ICIs for the treatment of digestive system cancers have not been systematically evaluated. Given the increasing use of ICI-based immunotherapies in patients with digestive system cancers shortly, clinicians must have a comprehensive understanding of the toxicity associated with these therapies. Here, we aim to provide clinicians and clinical pharmacists with an evidence-based basis for immunosuppression in the treatment of gastrointestinal tumors by conducting a systematic evaluation and meta-analysis of published randomized controlled trials in the field of the digestive system regarding the trAEs of ICI.

## Methods

### Search strategy and selection criteria

We did a systematic review and meta-analysis to identify published RCTs using ICI therapy for digestive system cancers that reported treatment-related adverse events. Papers published between January 1, 2010, and May 18, 2022, were searched in PubMed, Embase, Web of Science, and Cochrane databases for the subject terms “Immune Checkpoint Inhibitors”, “PD-1”, “PD-L1”, “CTLA-4”,” Stomach Neoplasms”, “Esophageal Neoplasms”, “Colorectal Neoplasms”, “Liver Neoplasms”, “Gastroesophageal junction carcinoma”, “Appendiceal Neoplasms”, “Splenic Neoplasms”, “Pancreatic Neoplasms”, and “Randomized Controlled Trials” (**Supplementary Table 1**). Relevant references, related reviews, and article references were also checked manually to avoid missing relevant articles. Two researchers (Kou Liqiu, Xie Xiaolu) conducted the literature search and data extraction independently. All conflicts were resolved by discussion with a third partner (Chen Xiu) to reach a consensus.

This study was done in accordance with the guidance of the PRISMA statement.

We used the following selection criteria: (1) Studies of randomized controlled trials which were published before May 18, 2022. (2) Participants diagnosed with digestive system (colorectal, liver, stomach, esophagus, gastroesophageal junction, pancreatic, spleen, appendix) cancers who were treated with at least one PD-1, PD-L1, or CTLA-4 inhibitor (e.g., Nivolumab, Pembrolizumab, Atezolizumab, Ipilimumab, Camrelizumab, etc.). (3) Clinical trials that report overall incidence or tabular data for trAE or irAE profiles, and (4) Studies published in English. Exclusion criteria: (1) Received treatment other than PD-1, PD-L1, or CTLA-4 inhibitors (e.g., chemotherapy, targeted therapy drugs); (2) Repeat publications (only the most recent publications were retained); (3) Case reports, letters, conference abstracts, animal studies, reviews, expert opinions, etc.

### Data extraction

Two investigators (Kou Liqiu, Wen Qinglian) independently obtained the following basic information from each included study: first author, year of publication, country, clinical trial number, cancer classification, median age, and enrollment, outcomes (total number of patients participating in safety analysis, number of patients discontinued and died due to treatment or immune-related AEs). AE terms are coded according to the Medical Dictionary of Regulatory Activities, and severity is graded according to the National Cancer Institute Common Terminology Criteria for Adverse Events. In this study, those AEs described as being of special interest were also extracted as irAE.

### Risk of bias and quality assessments

The Cochrane Collaboration tool was used to assess the risk of bias in the RCTs that were part of our study. Each trial was judged to be at low, unclear, or high risk for random sequence generation, allocation concealment, blinding of participants, personnel, and outcome assessors, incomplete outcome data, and selective outcome reporting. Two authors (Kou Liqiu, Chen Xiu) independently performed this process, and disagreements in ratings were resolved by a third investigator (Xie Xiaolu).

### Statistical analysis

We performed a meta-analysis of the overall incidence of ICI for the treatment of digestive system cancers. The primary study was the incidence of AEs, which was calculated by dividing the number of AEs by the total number of patients, the summary measure of the primary outcome was the incidence (95% CI). Before the meta-analysis, the incidence was logit converted and classic correction of 0.5 was added to zero events. Additionally, Subgroup analyses of AE incidence were performed according to type of cancer, type of combination of single ICI or dual ICI, and type of different agent. Multivariate multilevel meta-analysis models were performed to assess risk factors for AE, with the primary outcome of interest being the overall AE incidence. The putative predictors evaluated, including cancer type, combination type, type of ICI agent, were chosen as moderators. Effect sizes for all comparative analyses were assessed using the odds ratio (95%). The χ2 test and I^2^ statistic were applied to estimate between-study heterogeneity. Significant heterogeneity was indicated for the χ2 test p<0.10 or I^2^>50%, and the random effects model was applied to the combined analysis. Otherwise, we applied a fixed-effects model. A random effects model was applied to the pooled analysis of the odds ratio. Statistical significance was considered when p<0.05. Publication bias detection was performed by Egger. If there was significant publication bias, pruning and filling were used to verify the robustness of the meta-analysis results. All analyses were done using SPSS statistical software (version 26.0) and R software (version 4.2.0).

## Results

### Search results

**Figure 1:**
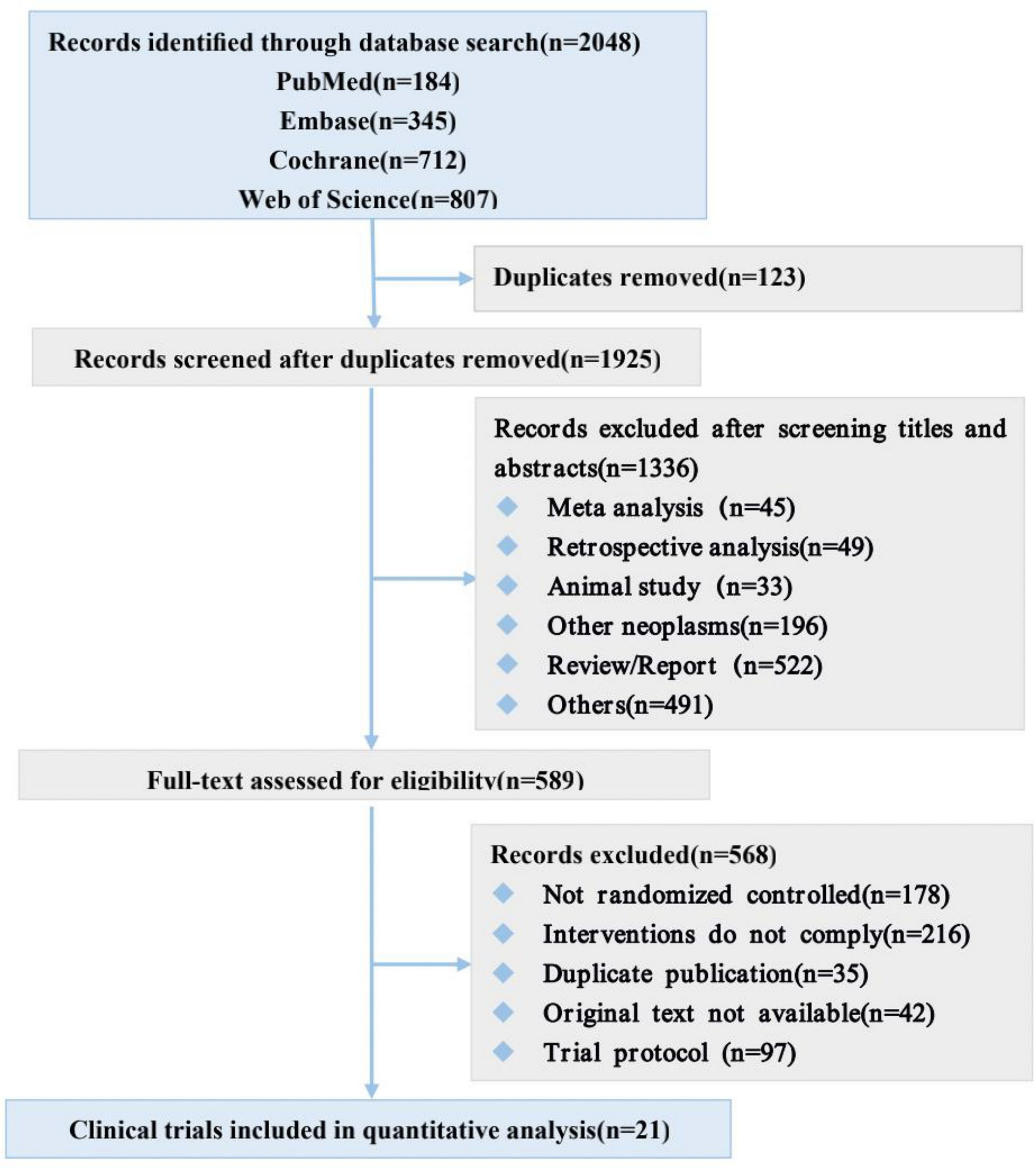
Study selection.

Our systematic search of PubMed, Embase, Web of Sciences, and Cochrane databases identified 2048 records (**Figure 1**), from which we selected 21 eligible studies involving 7108 patients for quantitative analysis. The main characteristics of the included studies are shown in **Table 1** and Main characteristics of ICI arms included in the meta-analysis for AEs comparison(**Supplementary Table 2**).

**Table 1:** Characteristics of studies included in the Meta-analysis.

Study	Year	Country	Trials identifier	Cancer type	Agents in arms	No.of.arms	Median age	Enrollment
Eng C (22)	2019	North,America,Europe, Asia,et al	NCT02788279	Colorectal Cancer	Atezolizumab+Cobimetinib *vs*. Atezolizumab *vs*. Regorafenib	1	56 (51–64)	363
André T (23)	2020	Asia,Western Europe, North America,et al	NCT02563002	Colorectal Cancer	Pembrolizumab *vs*. Chemotherapy	1	63 (24–93)	307
Chen EX (24)	2020	Canada	NCT02870920	Colorectal Cancer	Tremelimumab+Durvaluma *vs*.BSC	1	65 (39-87)	180
Hu H (25)	2022	China	NCT03926338	Colorectal Cancer	Toripalimab *vs*. Toripalimab+Celecoxib	1	53 (45–60)	34
Finn RS (26)	2020	Argentina,Australia, Canada,et al	NCT02702401	Liver cancer	Pembrolizumab *vs*.Placebo	1	67 (18-91)	413
Kelley RK (27)	2021	Japan	NCT02519348	Liver cancer	Tremelimumab+Durvaluma	4	66 (26-86)	332
Yau T (28)	2022	United States, Canada,Europe,et al	NCT02576509	Liver cancer	Nivolumab *vs*. Sorafenib	1	65 (19–89)	743
Kaseb AO (29)	2022	African-American, Asian	NCT03222076	Liver cancer	Nivolumab *vs*. Nivolumab+Ipolimumab	2	64 (56–68)	27
Qin S (30)	2020	China	NCT02989922	Liver cancer	Camrelizumab *vs*. Camrelizumab	2	48 (41–56)	303
Lee MS (31)	2020	Asian,Hawaiian, African American,et al	NCT02715531	Liver cancer	Atezolizumab+Bevacizumab *vs*.Atezolizumab	1	63 (23–85)	104
Yau T (32)	2020	United States, Italy,Spain,et al	NCT01658878	Liver cancer	Nivolumab+Ipolimumab	3	60 (52.5-66.5)	148
Shah MA (33)	2021	America,Europe,Australian	NCT02864381	Gastric Cancer	Nivolumab *vs*. Andecaliximab	1	62 (23–80)	144
Shitara K (34)	2020	Europe,North,America, Australian,et al	NCT02494583	Gastric Cancer	Pembrolizumab *vs*. Pembrolizumab+Chemotherapy vs.Chemotherapy+Placebo	1	62 (20-87)	763
Shitara K (35)	2018	Europe, Israel, North America,et al	NCT02370498	Gastroesophageal junction cancer	Pembrolizumab *vs*. Paclitaxel	1	62·5 (54–70)	592
Satoh T (36)	2019	Japan,Korea,Taiwan. China	NCT02267343	Gastroesophageal junction cancer	Nivolumab *vs*. Placebo	1	62 (20–83)	493
Kelly RJ (37)	2020	Europe,United States,Canada,et al	NCT02743494	Gastroesophageal junction cancer	Nivolumab *vs*. Placebo	1	62 (26–82)	792
Chung HC (38)	2022	China,Malaysia,Korea	NCT03019588	Gastroesophageal junction cancer	Pembrolizumab *vs*. Paclitaxel	1	61 (32- 75)	94
Bang YJ (39)	2017	Asia,Row,et al	NCT01585987	Gastroesophageal junction cancer	Ipilimumab *vs*. BSC	1	65 (34–86)	143
Kojima T (40)	2020	Argentina,Australia, Brazil,et al	NCT02564263	Esophageal cancer	Pembrolizumab *vs*. Chemotherapy	1	63 (23-84)	628
Park S (41)	2022	Korea	NCT02520453	Esophageal cancer	Durvaluma *vs*. Placebo	1	64 (39-76)	86
Kato K (42)	2019	China,Denmark, Germany,et al	NCT02569242	Esophageal cancer	Nivolumab *vs*.Chemotherapy	1	64 (57–69)	419

BSC, best supportive care.

### Risk of bias assessment

Two independent reviewers (Kou Liqiu, Xie Xiaolu) assessed the quality of evidence from 21 studies of RCTs using the Cochrane Collaboration’s tool. The overall risk of bias was low in the included studies. 2 studies did not perform allocation concealment and 8 studies did not provide relevant information. 1 study did not conceal it from patients and staff. 3 studies were not blinding of outcome assessment. In addition, 13 studies were open-label studies and therefore may have had some publication bias. The risk of bias status is summarized in (**Supplementary Table 3**).

### Studies evaluating the incidence of trAEs and irAEs

**Figure 2:**
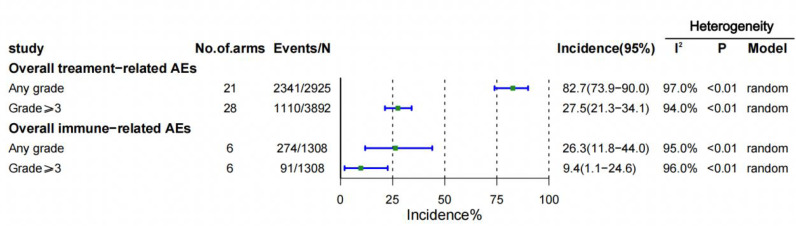
Overall incidences of treatment-related and immune-related adverse events (AEs). CI = confidence interval.

A total of 21 (22–42) trials were included, all reporting the incidence of trAEs and 6 (23, 26, 34–36, 39) trials reporting the incidence of irAEs. The overall incidence of trAEs of any grade was 82.7% (95% CI 73.9-90.0) in the 21 (22–27, 29, 30, 32, 34–40) study arms, and the incidence of trAEs of grade 3 or higher was 27.5% (95% CI 21.3-34.1) in the 28 (22–42) study arms. There were 6 (23, 26, 34–36, 39) arms of the study reported irAEs of any grade, with an overall incidence of irAEs of any grade was 26.3% (95% CI 11.8-44.0), respectively, and 6 (23, 26, 34–36, 39) arms of the study reported irAEs of grade 3 or higher, and the incidence was 9.4% (95% CI 1.1-24.6) (**Figure 2**).

### Assessment of the occurrence profile of AEs

We performed a pooled analysis of the incidence of AEs in ICI for the treatment of digestive system cancers. The most common trAEs of any grade were hypoalbuminemia (79.7% [95% CI 72.3-87.0]), lactate dehydrogenase increase (77.1% [95% CI 69.4-84.8]), lymphopenia (72.0% [95% CI 63.8-80.3]). (**Supplementary Figure 1**). We only reported trAEs of any grade with an incidence of 10% or more. TrAEs with grade 3 or higher were most common for lymphopenia (22.0% [95% CI 14.4-29.6]), lactate dehydrogenase increase (16.9% [95% CI 10.1-23.8]), hyponatremia (16.5% [95% CI 11.1-21.8]). (**Supplementary Figure 2**).

Among the adverse events associated with irAEs, the most common in any class were rash (26.4% [95% CI 19.2-33.5]), hypothyroidism (9.5% [95% CI 7.6-11.4]), diarrhea (6.8% [95% CI 3.3-10.3]). (**Supplementary Figure 3**). The incidence of irAEs of grade 3 or higher was lower, with the most common being diarrhea (5.4% [95% CI 2.3-5.1]), rash (5.1% [95% CI 0.7-9.4]), hepatitis (4.1% [95% CI 2.6-5.7]). (**Supplementary Figure 4**).

### TrAEs incidence by cancer type, combination types(single- or two-drug), and type of ICI agent

**Figure 3:**
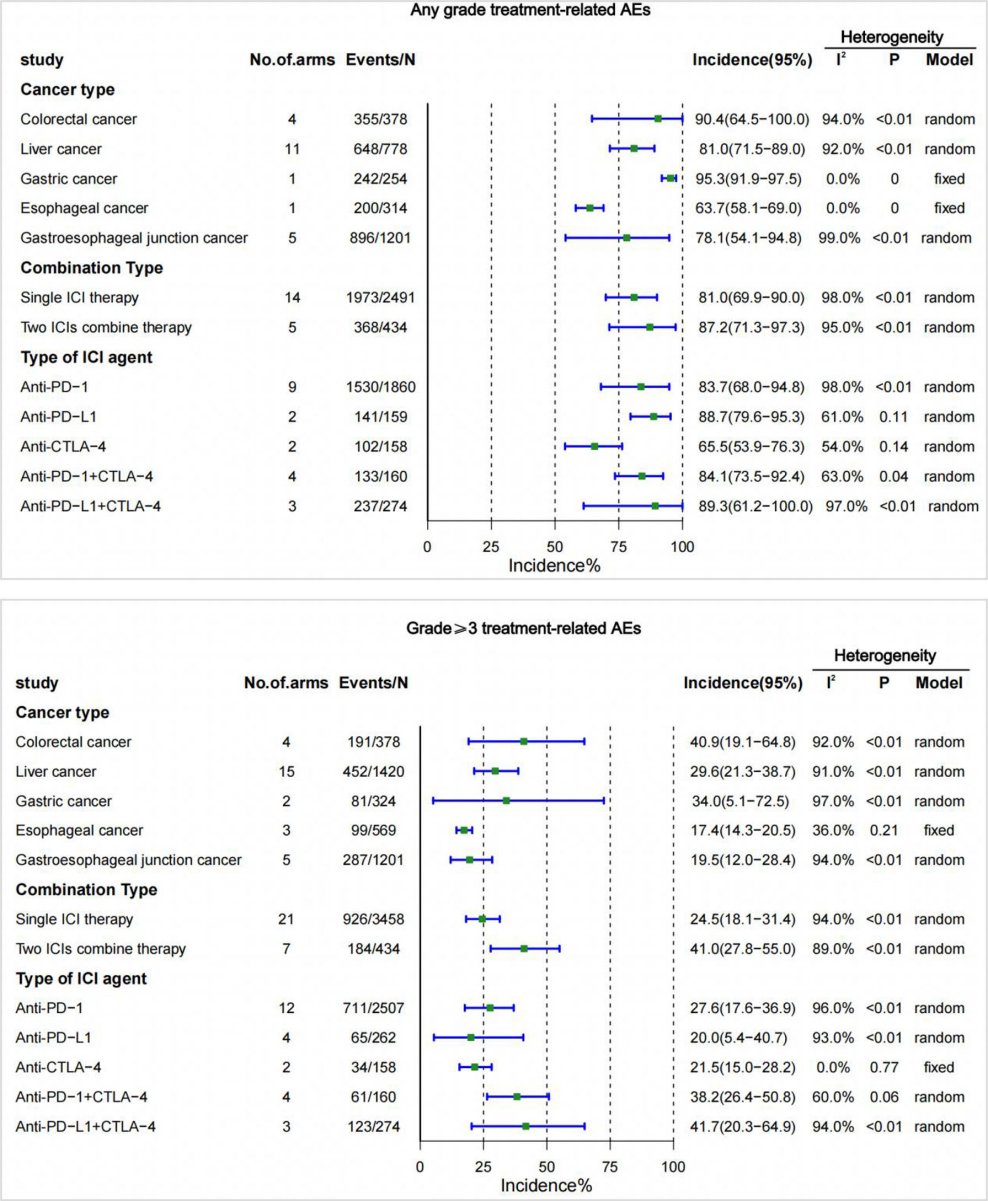
Incidence of treatment-related adverse events (trAEs) by cancer type, single- or two-drug combinations, and type of ICI agent. CI, confidence interval; ICI, immune checkpoint inhibitor.

In the analysis of the incidence of trAEs in different cancer types, we found that gastric cancer had the highest incidence of trAEs of any grade (95.3% [95% CI 91.9-97.5]), followed by colorectal cancer (90.4% [95% CI 64.5-100.0]), liver cancer (81.0% [95% CI 71.5-89.0]), gastroesophageal junction cancer (78.1% [95% CI 54.1-94.8]), and the lowest incidence was esophageal cancer (63.7% [95% CI 58.1-69.0]). Among the incidence rates of grade 3 or higher, colorectal cancer (40.9% [95% CI 19.1-64.8]) had the highest incidence rate, followed by gastric cancer (34.0% [95% CI 5.1-72.5]), liver cancer (29.6% [95% CI 21.3-38.7]), gastroesophageal junction cancer (19.5% [95% CI 12.0-28.4]), and esophageal cancer (17.4% [95% CI 14.3-20.5]) had the lowest incidence (**Figure 3**).

In different combination types(single- or two-drug), trAEs was higher with two ICIs combined than with ICIs alone. The incidence of pooling of any grade was 81.0%, and the incidence of grade 3 or higher was 24.5% when treated with a single ICI drug. The incidence of any grade was 87.2%, and the incidence of grade 3 or higher was 41.0% with the combination of two ICIs (**Figure 3**).

Among the different types of ICI agents, the highest incidence of trAEs for any grade of anti-PD-L1 (88.7% [95% CI 79.6-95.3]), and the highest incidence of trAEs for grade 3 or higher was for anti-PD-1 (27.6% [95% CI 17.6-36.9]). The lowest incidence of trAEs of any grade (65.5% [95% CI 53.9-76.3]) was anti-CTLA-4, and the lowest incidence of grade 3 or higher was anti-PD-L1 (20.0% [95% CI 5.4-40.7]). Additionally, the different types of ICI combined (PD-1/PD-L1+CTLA-4) increased the risk of trAEs. The risk of the combination of anti-PD-L1 and anti-CTLA-4 is most evident in trAEs of any grade (89.3% [95% CI 61.2-100.0]) and trAEs of grade 3 or higher(41.7%[95%CI20.3-64.9]) (**Figure 3**).

### IrAEs incidence by cancer type, single- or two-drug(PD-1/PD-L1+CTLA-4) combinations, and type of ICI agent

**Figure 4:**
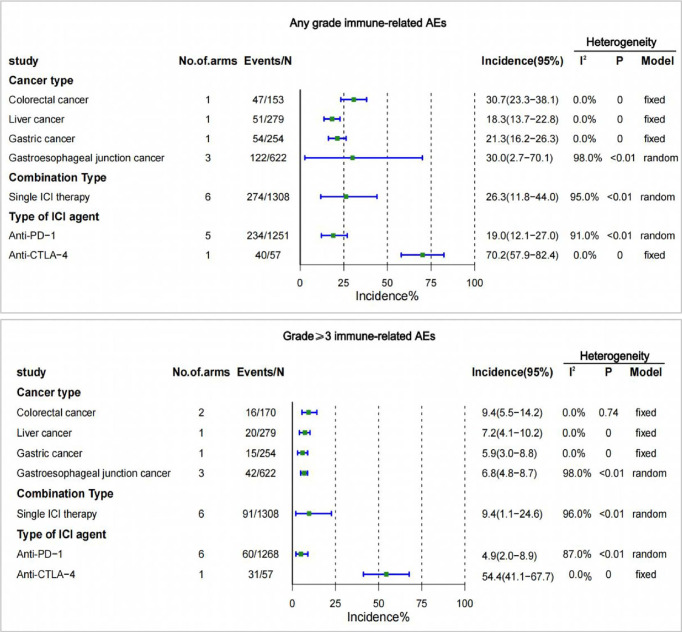
Incidence of treatment-related adverse events (irAEs) by cancer type, single- or two-drug combinations, and type of ICI agent. CI, confidence interval; ICI, immune checkpoint inhibitor.

There were no trials reporting irAEs for esophageal cancer in the included studies. Colorectal cancer had the highest incidence of irAEs at 30.7% for any grade, and 9.4% for grade 3 or higher. The lowest incidence of irAEs at any grade was for liver cancer (18.3%), and the lowest incidence of irAEs at grade 3 or higher was for gastric cancer (5.9%) (**Figure 4**).

The incidence of irAEs with two ICI combine therapy was not reported in the included studies. There were 6 studies reporting irAEs of any grade and irAEs of grade 3 or higher. The incidence of irAEs of any grade with single-agent ICI was 26.3% and the incidence of grade 3 or higher irAEs was 9.4% (**Figure 4**).

The occurrence of irAEs was reported only in the anti-PD-1 and anti-CTLA-4 treatment groups among the different types of ICI drugs. The incidence of irAEs was higher for anti-CTLA-4 of any grade (70.2% [95% CI 57.9-82.4]), and for grade 3 or higher (54.4% [95% CI 41.1-67.7]) than for anti-PD-1 (**Figure 4**).

### Multivariable regression analysis

Colorectal cancer has a significantly higher risk of trAEs and irAEs than most other cancers of the digestive system. However, it is noteworthy that the incidence of trAEs at any grade was higher in gastric cancer than in colorectal cancer (OR:2.59,95% CI:1.34-5.01, P=0.0048). Two ICI drug therapy was associated with an increased risk of trAEs compared with a single ICI drug therapy. Combination therapy with two ICIs was associated with an increased risk of trAEs of any grade (OR:1.46, 95% CI:1.11-1.94, P=0.0075) and grade 3 or higher (OR:3.72, 95% CI:3.03-4.56, P<0.0001).

In addition, different types of ICI drugs are associated with trAEs and irAEs. We found that CTLA-4 had the lowest incidence of trAEs (OR: 0.39, 95% CI: 0.28-0.56, P<0.0001 for any grade and OR:1.55,95% CI:1.05-2.88, P=0.0257 for grade ≥3), but the highest incidence of irAEs (OR: 10.23, 95% CI: 5.70-18.36, P<0.0001 for any grade and OR: 23.19, 95% CI: 12.98-41.44, P<0.0001 for grade ≥3). Furthermore, the combination of PD-1 and CTLA-4 immune checkpoint inhibitors was associated with a higher risk of trAEs (OR:4.60, 95% CI: 3.58-5.92, P<0.0001 for grade≥3). The evaluation results are shown in (**Table 2**).

**Table 2:** Analysis of factors associated with the occurrence of treatment-related adverse events (trAEs) and immune-related adverse events (irAEs).

Variables	trAES						irAEs					
	Any grade			Grade≥3			Any grade			Any grade		
	OR	95%CI	P value	OR	95%CI	P value	OR	95%CI	P value	OR	95%CI	P value
**Cancer type**												
Colorectal cancer	Referent			Referent			Referent			Referent		
Liver cancer	0.64	(0.44-0.93)	0.0177	0.46	(0.36-0.58)	<0.0001	0.50	(0.32-0.8)	0.0034	0.74	(0.37-1.48)	0.40
Gastric cancer	2.59	(1.34-5.01)	0.0048	0.33	(0.24-0.45)	<0.0001	0.61	(0.39-0.96)	0.0032	0.60	(0.29-1.26)	0.1777
Esophageal cancer	0.23	(0.15-0.33)	<0.0001	0.21	(0.15-0.28)	<0.0001	0.55	(0.37-0.82)	0.0031	0.70	(0.38-1.27)	0.2403
Gastroesophageal junction cancer	0.38	(0.27-0.53)	<0.0001	0.31	(0.24-0.39)	<0.0001	Na	Na	Na	Na	Na	Na
**Combination Type**												
Single ICI therapy	Referent			Referent			Referent			Referent		
Two ICI combine therapy	1.46	(1.11-1.94)	0.0075	3.72	(3.03-4.56)	<0.0001	Na	Na	Na	Na	Na	Na
**Type of ICI agent**												
Anti–PD-1	Referent			Referent			Referent			Referent		
Anti–PD-L1	1.69	(1.02-2.80)	0.0417	1.87	(1.39-2.5)	<0.0001	Na	Na	Na	Na	Na	Na
Anti–CTLA-4	0.39	(0.28-0.56)	<0.0001	1.55	(1.05-2.88)	0.0257	10.23	(5.70-18.36)	<0.0001	23.19	(12.98-41.44)	<0.0001
Anti–PD-1+CTLA-4	1.38	(0.96-1.99)	0.0837	4.60	(3.58-5.92)	<0.0001	Na	Na	Na	Na	Na	Na
Anti–PD-L1+CTLA-4	1.06	(0.69-1.63)	0.7827	3.48	(2.51-4.84)	<0.0001	Na	Na	Na	Na	Na	Na

Na, Not available.

### Assessment of the occurrence profile of single and two-ICI AEs

**Figure 5:**
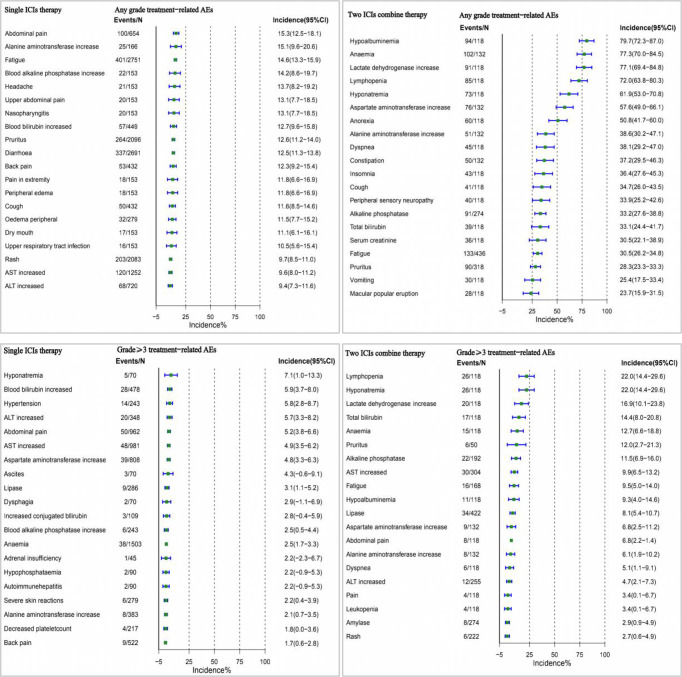
Overview of single and two ICI AEs.

In a multivariate analysis, we found that the incidence of AEs was higher with two drug therapy than with monotherapy. Therefore, we performed a comprehensive analysis of this influencing factor, and the top three incidences of monotherapy of any grade of AEs occurred were abdominal pain, alanine aminotransferase increase, and fatigue, while two ICIs were hypoalbuminemia, anaemia, and lactate dehydrogenase increase. Among AEs of grade 3 or higher, the highest incidence of single agents was hyponatremia, blood bilirubin increased, and hypertension, and the two ICIs were lymphopenia, hyponatremia, and lactate dehydrogenase increase (**Figure 5**).

### Assessment of the occurrence profile of different types of ICI agents AEs

Among adverse reactions of any grade, the most common adverse reactions to anti-PD-1 were maculopapular rash (30.8% [95% CI 1.7-59.8]), fatigue (19.2% [95% CI 17.1-21.2]), the most common adverse reactions to anti-PD-L1 were pruritus (10.8% [95% CI 4.7-16. 9]), hypothyroidism (9.9% [95% CI 4.0-15.8]), and the most common adverse reactions to anti-CTLA-4 were constipation (37.9% [95% CI 29.5-46.3]), cough (34.7% [95% CI 26.0-43.5]), while the most common adverse reaction to different types of ICI combination therapy with anti-PD-1 + anti-CTLA-4 was aspartate aminotransferase increased (50.0% [95% CI 20.0-80.0]), alanine aminotransferase increased (50.0% [95% CI 20.0-80.0]), and the most common adverse reactions of anti-PD-L1+anti-CTLA-4 were anemia (83.9% [95% CI 77.2-90.6]), hypoalbuminemia (79.7% [95% CI 72.3- 87.0]).

Of the trAEs of grade 3 or higher, the most common adverse reactions to anti-PD-1 were blood bilirubin increase (7.5% [95% CI 4.4-10.6]), hypertension (7.2% [95% CI 5.3-11.3]), anti-PD-L1 most common adverse reactions were abdominal pain (4.4% [95% CI 0.1-8.8]), aspartate aminotransferase increase (3.4% [95% CI 1.4-8.3]), anti-CTLA-4 the most common adverse reactions were diarrhea (8.7% [95% CI 3.7-13.7]), AST increase (8.7% [95% CI 1.9-15.5]) and anti-PD-1+ anti-CTLA-4 the most common adverse reactions were aspartate aminotransferase increase (28.6% [95% CI 1.5-55.6]), and alanine aminotransferase (28.6% [95% CI 1.5-55.6]), and the most common adverse reactions to anti-PD-L1+anti-CTLA-4 were lymphopenia (22.0% [95% CI 14.4-29.6]) and hyponatremia (22.0% [95% CI 14.4-29.6]).

The most common adverse effects of anti-PD-1 in irAEs of any grade were hypothyroidism (9.0% [95% CI 6.9-11.1]), hyperthyroidism (4.1% [95% CI 2.7-4.6]), and anti-PD-1 + anti-CTLA-4 were rash (26.4% [95% CI 19.2-33.5]), hypothyroidism (11.2% [95% CI 6.9-15.6]). Among irAEs of grade 3 or higher, the most common adverse reactions to anti-PD-1 were hepatitis (1.8% [95% CI 0.6-3.0]), adrenal insufficiency (1.3% [95% CI -0.5-3.1]), while the most common adverse reactions to anti-PD-1+ anti-CTLA-4 were fatigue (3.5% [95% CI -1.4-8.4]), diarrhea (5.4% [95% CI 2.3-8.5]) (**Supplementary Figures 5-18** summarizes the 20 most common AEs reported for different ICI agent types).

### Publication bias

We tested for publication bias in the occurrence profiles of treatment-related and immune-related adverse events of any grade and grade 3 or higher, and publication bias was present in all except for trAEs published at grade 3 or above (p=0.056). We modified the funnel plot by the pruning and filling method, and the results were still biased. We analyzed the following reasons: (1) We included five types of digestive system cancers, and there were differences in the responsiveness of different cancers to ICI drugs. (2) Different types of drug combinations may also make a difference in the occurrence of adverse events (**Supplementary Figure 19**).

## Discussion

This systematic evaluation and meta-analysis included 21 randomized controlled trials examining the incidence and profile of adverse events associated with immune checkpoint inhibitor-based therapies for digestive system cancers. Overall, our pooled analysis reported 82.7% and 27.5% incidence of trAEs of any grade and grade 3 or higher. These results showed a reduction in the incidence of trAEs compared with the incidence reported in previous studies of risk across cancer types(including cancer of the gastric or gastroesophageal junction) (43). In previous meta-analysis, the trAEs of different ICI in various cancer types were analyzed, with 86.8% and 35.9% of trAEs of any grade and grade 3 or higher, respectively, but the incidence of irAEs was not systematically evaluated in that article (43). However, some of the irAEs may be severe and lead to permanent disease (44). Hence, the incidence of irAEs was pooled in our meta-analysis and there were 26.3% and 9.4% of irAEs of any grade and grade 3 or higher. So it’s necessary to focus on irAEs to reduce potential short- and long-term complications and optimize quality of life and long-term outcomes.

There were some interesting findings from multivariate analysis. First, the incidence of AE subgroups based on cancer type had the highest risk of trAEs and irAEs in colorectal cancer. That may be due to the following reasons: while ICI therapy has shown an unusually high depth and frequency of durable response in clinical trials in patients with mismatch repair-deficient(MMR-D) colorectal cancer with much fewer treatment-related adverse events (45, 46), whereas MMR occurs more frequently in early-stage tumors than in late-stage tumors (47), two of the four colorectal cancer studies included in this study were in advanced MMR-D colorectal cancer and one was metastatic colorectal cancer; In addition, Venderbosch et al. found that the incidence of dMMR in metastatic colorectal cancer was only 5%, which was lower than that of early-stage colorectal cancer (19.72%) (48). Second, ICI combination therapy(single or two drugs) was associated with an increased risk of trAEs, more pronounced in trAEs of any grade. That’s consistent with the findings of Janjigian YY (49), Gubens MA (50), et al. Although the incidence of trAEs was higher with the combination of two ICIs than with a single ICI, it was still lower or similar to conventional treatment (chemotherapy and targeted therapy) (51, 52), thus, they can be managed appropriately through close monitoring and early recognition of relevant signs and symptoms. The included ICI combination therapy trials did not report the occurrence of irAEs, which may be due to the fact that most of the pilot studies focused on efficacy. Thirdly, Across the different types of ICI drugs, our meta-analysis found that anti-CTLA-4 had the lowest incidence of trAEs but the highest incidence of irAEs (53), and increased the risk of trAEs when combined with PD-1, which was consistent with the study by Osipov A et al (54). Therefore, TRAE, especially TRAE of grade 3 or higher, becomes one of the major issues that cannot be ignored in combination therapy. Take special care of lymphopenia when using PD-1 and CTLA-4. Lymphopenia is a predictive indicator and has a significant impact on survival. It is usually used with the addition of steroids (55).

Immune-related adverse reactions (irAEs) are immune activations caused by regulatory T-cell activity that can cause immune-related adverse reactions, resulting in symptoms associated with the corresponding organs (56). The most common irAEs include rash, colitis, hepatitis, endocrine disorders, and pneumonia (57). In this study, the most common incidence of irAEs at any level was found to be rash, hypothyroidism, and diarrhea, which is generally similar to the study by Wu, et al (17). However, Wu et al. studied the incidence of adverse events in ICI for urologic cancers, suggesting that irAEs are primarily drug-related and do not differ significantly concerning the type of cancer. In addition, grade 3 or higher irAEs are most commonly associated with diarrhea, rash, and hepatitis. Hepatitis is potentially fatal toxicity and immune-related hepatitis has a good prognosis but often requires treatment discontinuation, high-dose steroids, and second-line immunosuppression (58, 59). Most irAEs can be controlled and reversed by discontinuing dosing or using corticosteroids (60), With infliximab, for example, most of the adverse immune system reactions are eliminated with proper management (57).

This study has several advantages: 1) This systematic evaluation and meta-analysis included 21 randomized controlled trials that examined the incidence and distribution of adverse events associated with immune checkpoint inhibitor therapies for digestive system cancers. 2) The AEs associated with ICI in digestive system cancers were summarized by meta-analysis, which found that the incidence, characteristics, and distribution of AEs varied by cancer type, combination therapy modality, and different types of agents. 3) A comprehensive analysis of adverse events in ICI combination therapy can be used as an early identification to provide patients with effective interventions to reduce their severity. It may provide a clinical reference and may contribute to clinical practice. However there are some limitations: (1) We included only clinical randomized controlled trials, limiting the generalizability of our results to the general population of patients in real-world settings. (2) We found considerable heterogeneity when performing the test for heterogeneity, and despite doing subgroup analyses, we were still unable to find significant sources of heterogeneity, and some unobserved confounding factors may hinder the interpretation of the overall incidence in each subgroup, so the results need to be treated with caution. (3) Adverse events were recorded in clinical trials using MedDRA, but in some cases, the definitions of MedDRA overlapped. For example, patients with liver symptoms may be recorded as having hepatitis, autoimmune hepatitis, or elevated liver enzymes in a different clinical trial, thereby impeding knowledge of the actual incidence of adverse events. (4) Subgroup analysis revealed relatively small sample sizes for the Anti-PD-L1, Anti-CTLA-4, and Anti-PD-1 + CTLA-4 groups, so the results will have to be considered with caution and justified by a large sample of high-quality trials.

## Conclusion

This meta-analysis summarizes the profile of ICI-based treatment of common trAEs and irAEs in cancers of the digestive system. Different cancer types, combined treatment methods and different drug types are associated with incidence and AE characteristics, such a comprehensive analysis of adverse events in ICI combination therapy can be used as an early identification to provide effective interventions to reduce the severity of these patients. It may provide a clinical reference and may contribute to clinical practice. Further large-scale studies are needed to confirm our findings.

## Author contributions

YL, JL and QW have the concept of the study. LK and XC developed and implemented the search strategy. LK and XX independently screened the titles and abstracts of all retrieved records. LK and XC performed the data extraction. LK conducted the meta-analysis. LK wrote the draft manuscript. All authors contributed to the article and approved the submitted version.

## Funding

This study was supported by the Sichuan Provincial Department of Education (SCYG2020-04, SCYG2019-04).

## Acknowledgments

Thanks to all the authors of this article for their contributions to this article.

## Conflict of interest

The authors declare that the research was conducted in the absence of any commercial or financial relationships that could be construed as a potential conflict of interest.

## Publisher’s note

All claims expressed in this article are solely those of the authors and do not necessarily represent those of their affiliated organizations, or those of the publisher, the editors and the reviewers. Any product that may be evaluated in this article, or claim that may be made by its manufacturer, is not guaranteed or endorsed by the publisher.
