# Supplementary material for: Adverse events of immune checkpoint inhibitors for patients with digestive system cancers: A systematic review and meta-analysis
Source: Front Immunol. 2022 Oct 21;13:1013186. doi: 10.3389/fimmu.2022.1013186 (PMC9634077; doi:10.3389/fimmu.2022.1013186)
Supplement: Supplementary file 1 [file DataSheet_1.pdf]

Supplementary Table 1. Search strategy

PubMed	Searching Strategy	Results
#1	((Immune Checkpoint Inhibitors[MeSH Terms]) OR (Checkpoint Inhibitors, Immune[Title/Abstract])) OR (Immune Checkpoint Inhibitor[Title/Abstract])) OR (Checkpoint Inhibitor, Immune[Title/Abstract])) OR (Immune Checkpoint Blockers[Title/Abstract])) OR (Checkpoint Blockers, Immune[Title/Abstract])) OR (Immune Checkpoint Blockade[Title/Abstract])) OR (Checkpoint Blockade, Immune[Title/Abstract])) OR (Immune Checkpoint Inhibition[Title/Abstract])) OR (Checkpoint Inhibition, Immune[Title/Abstract])) OR (PD-L1 Inhibitors[Title/Abstract])) OR (PD L1 Inhibitors[Title/Abstract])) OR (PD-L1 Inhibitor[Title/Abstract])) OR (PD L1 Inhibitor[Title/Abstract])) OR (Programmed Death-Ligand 1 Inhibitors[Title/Abstract])) OR (Programmed Death Ligand 1 Inhibitors[Title/Abstract])) OR (PD-1-PD-L1 Blockade[Title/Abstract])) OR (Blockade, PD-1-PD-L1[Title/Abstract])) OR (PD 1 PD L1 Blockade[Title/Abstract])) OR (CTLA-4 Inhibitors[Title/Abstract])) OR (CTLA 4 Inhibitors[Title/Abstract])) OR (CTLA-4 Inhibitor[Title/Abstract])) OR (CTLA 4 Inhibitor[Title/Abstract])) OR (Cytotoxic T-Lymphocyte-Associated Protein 4 Inhibitors[Title/Abstract])) OR (Cytotoxic T Lymphocyte Associated Protein 4 Inhibitors[Title/Abstract])) OR (Cytotoxic T-Lymphocyte-Associated Protein 4 Inhibitor[Title/Abstract])) OR (Cytotoxic T Lymphocyte Associated Protein 4 Inhibitor[Title/Abstract])) OR (PD-1 Inhibitors[Title/Abstract])) OR (PD 1 Inhibitors[Title/Abstract])) OR (PD-1 Inhibitor[Title/Abstract])) OR (Inhibitor, PD-1[Title/Abstract])) OR (PD 1 Inhibitor[Title/Abstract])) OR (Programmed Cell Death Protein 1 Inhibitor[Title/Abstract])) OR (Programmed Cell Death Protein 1 Inhibitors[Title/Abstract])) OR (nivolumab[Title/Abstract])) OR (pembrolizumab[Title/Abstract])) OR	**28729**

	(atezolizumab[Title/Abstract])) OR (ipilimumab[Title/Abstract])) OR (camrelizumab[Title/Abstract])) OR (toripalimab[Title/Abstract])) OR (tislelizumab[Title/Abstract])) OR (sintilimab[Title/Abstract])) OR (durvalumab[Title/Abstract])) OR (envafolimab[Title/Abstract])) OR (cemiplimab[Title/Abstract])	
#2	((Stomach Neoplasms[MeSH Terms]) OR (Neoplasm, Stomach[Title/Abstract])) OR (Stomach Neoplasm[Title/Abstract])) OR (Neoplasms, Stomach[Title/Abstract])) OR (Gastric Neoplasms[Title/Abstract])) OR (Gastric Neoplasm[Title/Abstract])) OR (Neoplasm, Gastric[Title/Abstract])) OR (Neoplasms, Gastric[Title/Abstract])) OR (Cancer of Stomach[Title/Abstract])) OR (Stomach Cancers[Title/Abstract])) OR (Gastric Cancer[Title/Abstract])) OR (Cancer, Gastric[Title/Abstract])) OR (Cancers, Gastric[Title/Abstract])) OR (Gastric Cancers[Title/Abstract])) OR (Stomach Cancer[Title/Abstract])) OR (Cancer, Stomach[Title/Abstract])) OR (Cancers, Stomach[Title/Abstract])) OR (Cancer of the Stomach[Title/Abstract])) OR (Gastric Cancer, Familial Diffuse[Title/Abstract])) OR (Esophageal Neoplasms[MeSH Terms])) OR (Esophageal Neoplasm[Title/Abstract])) OR (Neoplasm, Esophageal[Title/Abstract])) undefined (Esophagus Neoplasm[Title/Abstract])) OR (Esophagus Neoplasms[Title/Abstract])) OR (Neoplasm, Esophagus[Title/Abstract])) OR (Neoplasms, Esophagus[Title/Abstract])) OR (Neoplasms, Esophageal[Title/Abstract])) OR (Cancer of Esophagus[Title/Abstract])) OR (Cancer of the Esophagus[Title/Abstract])) undefined (Esophagus Cancer[Title/Abstract])) OR (Cancer, Esophagus[Title/Abstract])) OR (Cancers, Esophagus[Title/Abstract])) OR (Esophagus Cancers[Title/Abstract])) OR (Esophageal Cancer[Title/Abstract])) OR (Cancer,	565208

	Esophageal[Title/Abstract])) OR (Cancers, Esophageal[Title/Abstract])) OR (Esophageal Cancers[Title/Abstract])) OR (Colorectal Neoplasms[MeSH Terms])) OR (Colorectal Neoplasm[Title/Abstract])) OR (Neoplasm, Colorectal[Title/Abstract])) OR (Neoplasms, Colorectal[Title/Abstract])) OR (Colorectal Tumors[Title/Abstract])) OR (Colorectal Tumor[Title/Abstract])) OR (Tumor, Colorectal[Title/Abstract])) OR (Tumors, Colorectal[Title/Abstract])) OR (Colorectal Cancer[Title/Abstract]))) OR (Cancer, Colorectal[Title/Abstract])) OR (Cancers, Colorectal[Title/Abstract])) OR (Colorectal Cancers[Title/Abstract])) OR (Colorectal Carcinoma[Title/Abstract])) OR (Carcinoma, Colorectal[Title/Abstract])) OR (Carcinomas, Colorectal[Title/Abstract])) OR (Colorectal Carcinomas[Title/Abstract])) OR (Liver Neoplasms[MeSH Terms])) OR (Neoplasms, Hepatic[Title/Abstract])) OR (Neoplasms, Liver[Title/Abstract])) OR (Liver Neoplasm[Title/Abstract])) OR (Neoplasm, Liver[Title/Abstract])) OR (Hepatic Neoplasms[Title/Abstract])) OR (Hepatic Neoplasm[Title/Abstract])) OR (Neoplasm, Hepatic[Title/Abstract])) OR (Cancer of Liver[Title/Abstract])) OR (Hepatocellular Cancer[Title/Abstract])) OR (Cancers, Hepatocellular[Title/Abstract])) OR (Hepatocellular Cancers[Title/Abstract])) OR (Hepatic Cancer[Title/Abstract])) OR (Cancer, Hepatic[Title/Abstract])) OR (Cancers, Hepatic[Title/Abstract])) OR (Hepatic Cancers[Title/Abstract])) OR (Liver Cancer[Title/Abstract])) OR (Cancer, Liver[Title/Abstract])) OR (Cancers, Liver[Title/Abstract])) OR (Liver Cancers[Title/Abstract])) OR (Cancer of the Liver[Title/Abstract])) OR (Cancer, Hepatocellular[Title/Abstract])) OR (Appendiceal Neoplasms[MeSH Terms])) OR (Appendiceal Neoplasm[Title/Abstract])) OR (Appendiceal Cancer[Title/Abstract])) OR (Appendix Cancer[Title/Abstract])) OR (Splenic Neoplasms[MeSH Terms])) OR (Splenic Neoplasm[Title/Abstract])) OR	

	(Spleen Neoplasms[Title/Abstract])) OR (Spleen Cancers[Title/Abstract])) OR (Splenic Cancer[Title/Abstract])) OR (Spleen Cancer[Title/Abstract])) OR (Pancreatic Neoplasms[MeSH Terms])) OR (Pancreatic Neoplasm[Title/Abstract])) OR (Pancreas Neoplasms[Title/Abstract])) OR (Pancreas Cancers[Title/Abstract])) OR (Pancreas Cancer[Title/Abstract])) OR (Pancreatic Cancer[Title/Abstract])	
#3	#1AND #2	**2479**
# 4	(((review[Title]) OR (meta[Title])) OR (meta-analysis[Title])) OR (review[Publication Type])	**3324946**
#5	#3 NOT #4	**1743**
#6	("2010/01/01"[Date - Publication] : "2022/05/18"[Date - Publication])	**14220285**
#7	#5 AND #6	**1686**
#8	#5 AND #6 Filters:Clinical Trial,Randomized Controlled Trial	**184**

Supplementary Table 2. Main characteristics of ICI arms included in the meta-analysis for AEs comparison

Study	Agents in arms	No. Of pts in safety analysis	All-grade trAEs,No	Grade≥3 trAEs,No	Discontinue	Death	All-grade irAEs,No	Grade≥3 irAEs,No	Discontinue	Death
Eng C 2019	Atezolizumab	90	Na	28	Na	Na	Na	Na	Na	Na
André T 2020	Pembrolizumab	153	13	0	Na	Na	47	14	Na	Na
Chen EX 2020	Tremelimumab+ Durvaluma	118	118	75	Na	Na	Na	Na	Na	Na
Hu H 2022	Toripalimab	17	Na	0	Na	Na	Na	2	Na	Na
Finn RS 2020	Pembrolizumab	279	269	145	48	1	51	20	40	0
Kelley RK 2021	Tremelimumab+ Durvaluma	74	61	28	Na	Na	Na	Na	Na	Na
	Tremelimumab	101	61	21	Na	Na	Na	Na	Na	Na
	Durvaluma	69	58	30	Na	Na	Na	Na	Na	Na
	Tremelimumab+ Durvaluma	82	58	20	Na	Na	Na	Na	Na	Na
Yau T 2022	Nivolumab	367	Na	82	16	Na	Na	Na	Na	Na
Kaseb AO 2022	Nivolumab	13	10	3	Na	Na	Na	Na	Na	Na
	Nivolumab+ Ipolimumab	14	12	6	Na	Na	Na	Na	Na	Na
Qin S 2020	Camrelizumab	109	Na	23	Na	Na	Na	Na	Na	Na
	Camrelizumab	108	Na	26	Na	Na	Na	Na	Na	Na
Lee MS 2020	Atezolizumab	58	Na	3	Na	Na	Na	Na	Na	Na
Yau T 2020	Nivolumab+ Ipolimumab	49	46	26	Na	Na	Na	Na	Na	Na
	Nivolumab+ Ipolimumab	49	37	14	Na	Na	Na	Na	Na	Na
	Nivolumab+ Ipolimumab	48	38	15	Na	Na	Na	Na	Na	Na
Shah MA 2021	Nivolumab	70	Na	38	Na	Na	Na	Na	Na	Na
Shitara K 2020	Pembrolizumab	254	242	43	10	3	54	15	4	1
Shitara K 2018	Pembrolizumab	294	155	42	Na	Na	61	10	Na	Na
Satoh T 2019	Nivolumab	271	144	44	Na	Na	21	1	Na	Na
Kelly RJ 2020	Nivolumab	532	510	183	68	Na	Na	Na	Na	Na
Chung HC 2022	Pembrolizumab	47	46	5	1	2	Na	Na	Na	Na

Bang YJ 2017	Ipilimumab	57	41	13	Na	Na	40	31	Na	Na
Kojima T 2020	Pembrolizumab	314	200	57	19	5	Na	Na	Na	Na
Park S 2022	Durvaluma	45	Na	4	Na	Na	Na	Na	Na	Na
Kato K 2019	Nivolumab	210	Na	38	8	2	Na	Na	Na	Na

Supplementary Table 3. Risk of bias and quality assessment in randomized controlled trials

Study	Random sequence generation	Allocation concealment	Blinding of paticipants and personnel	Blinding of outcome assessment	Incomplete outcome data	Selective reporting	Other bias
Eng C 2019	Low	Low	Low	Low	Low	Low	Low
André T 2020	Low	Low	Low	Low	Low	Low	Low
Chen EX 2020	Low	Unclear	Low	Low	Low	Low	Low
Hu H 2022	Low	Low	Low	Low	Low	Low	Low
Finn RS 2020	Low	Unclear	Unclear	Unclear	Low	Low	Low
Kelley RK 2021	Low	Unclear	Unclear	Low	Low	Low	Low
Yau T 2022	Low	Low	Low	Low	Low	Low	Low
Kaseb AO 2022	Low	Low	Low	High	Low	Low	Low
Qin S 2020	Low	High	High	High	Low	Low	Low
Lee MS 2020	Low	Low	Low	Low	Low	Low	Low
Yau T 2020	Low	Low	Low	Low	Low	Low	Low
Shah MA 2021	Low	Unclear	Low	Unclear	Low	Low	Low
Shitara K 2020	Low	Unclear	Low	Unclear	Low	Low	Low
Shitara K 2018	Low	High	Low	High	Low	Low	Low
Satoh T 2019	Low	Low	Low	Low	Low	Low	Low
Kelly RJ 2020	Low	Low	Low	Low	Low	Low	Low
Chung HC 2022	Low	Unclear	Low	Unclear	Low	Low	Low
Bang YJ 2017	Low	Unclear	Low	Unclear	Low	Low	Low
Kojima T 2020	Low	Unclear	Low	Unclear	Low	Low	Low
Park S2022	Low	Low	Low	Low	Low	Low	Low
Kato K 2019	Low	Low	Low	Low	Low	Low	Low

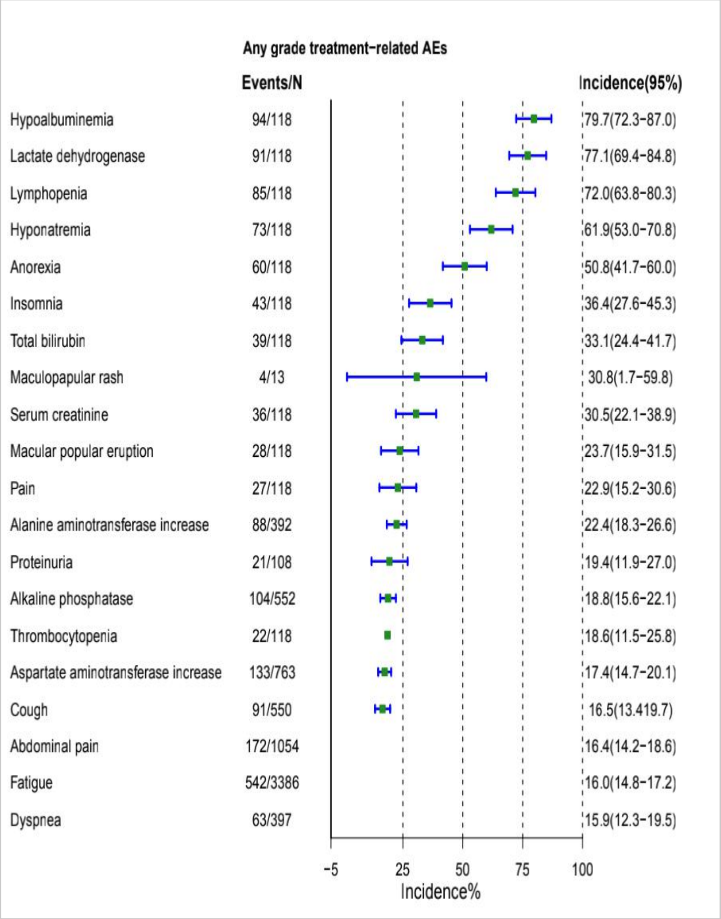

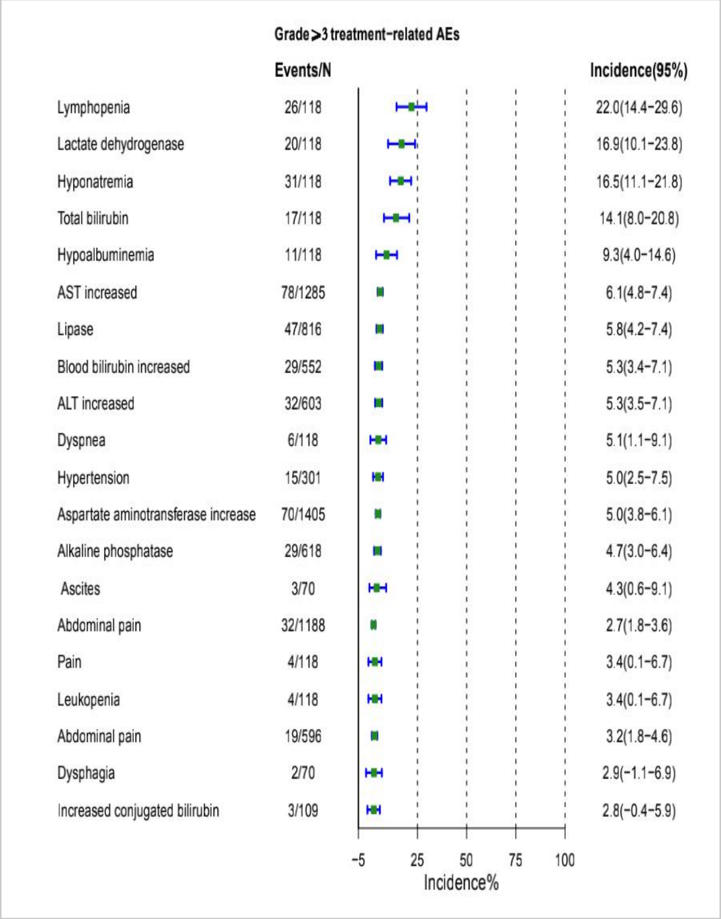

Supplementary Figure 1. Profile of trAEs of any grade

Supplementary Figure 2. Profile of trAEs of grade≥3

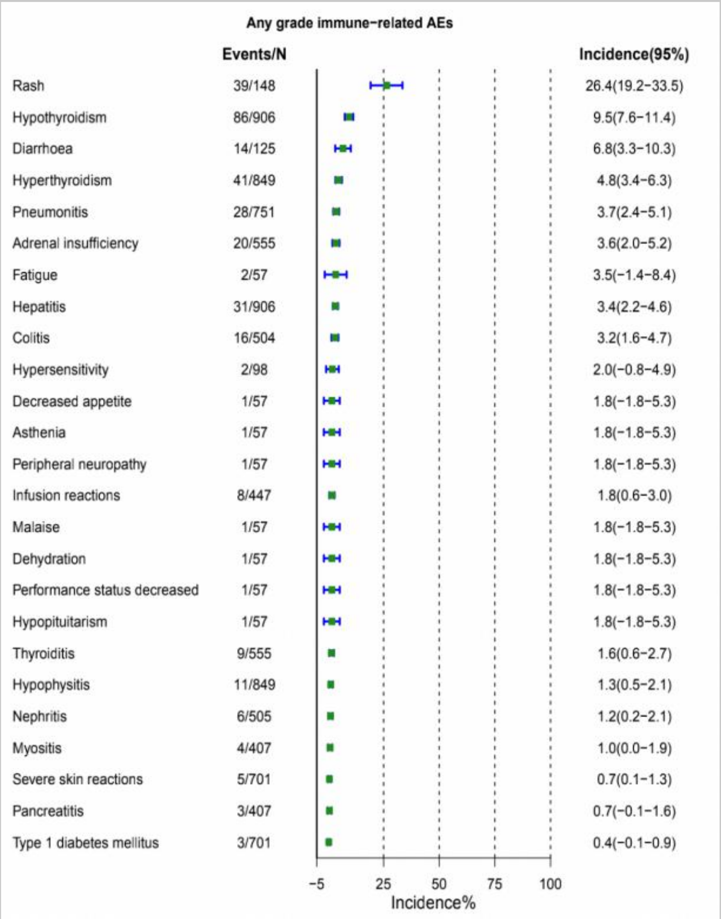

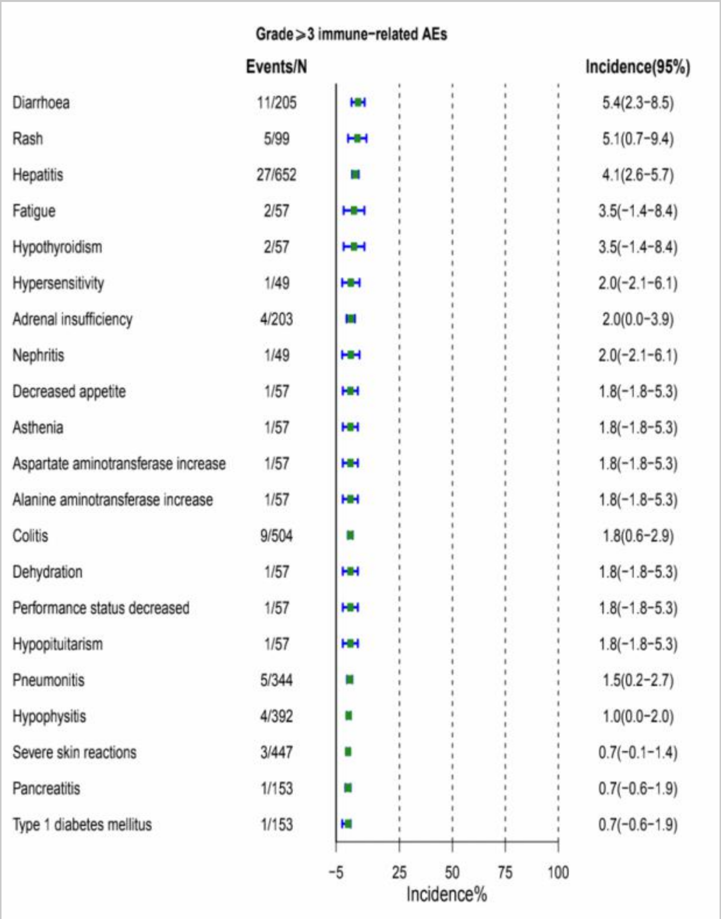

Supplementary Fig 3. Profile of irAEs of any grade

Supplementary Fig 4. Profile of irAEs of grade≥3

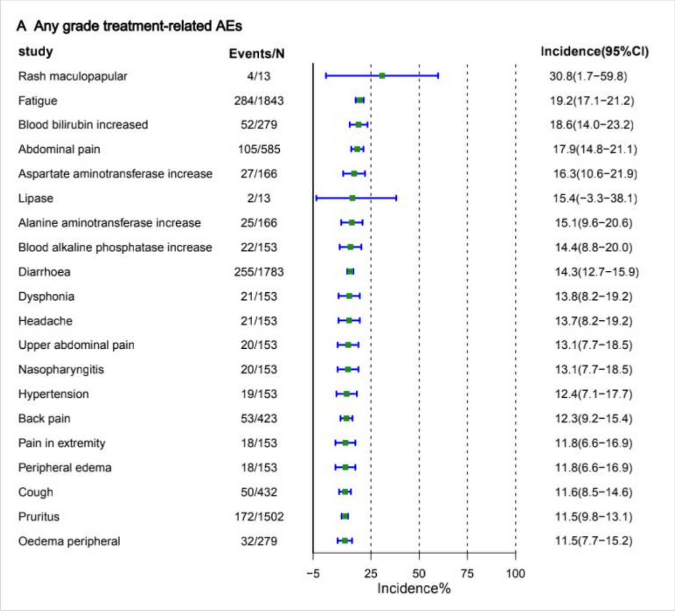

Supplementary Fig 5. Any grade of PD-1 inhibitor trAEs adverse events

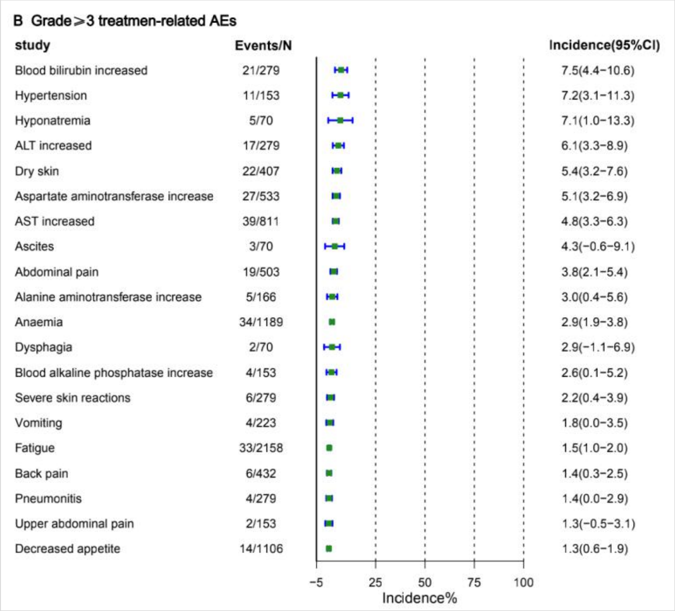

Supplementary Fig 6. Grade≥3 of PD-1 inhibitor trAEs adverse events

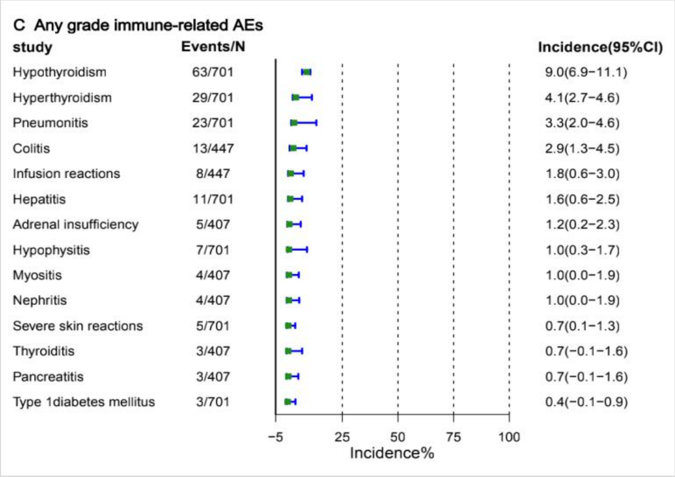

Supplementary Fig 7. Any grade of PD-1 inhibitor irAEs adverse events

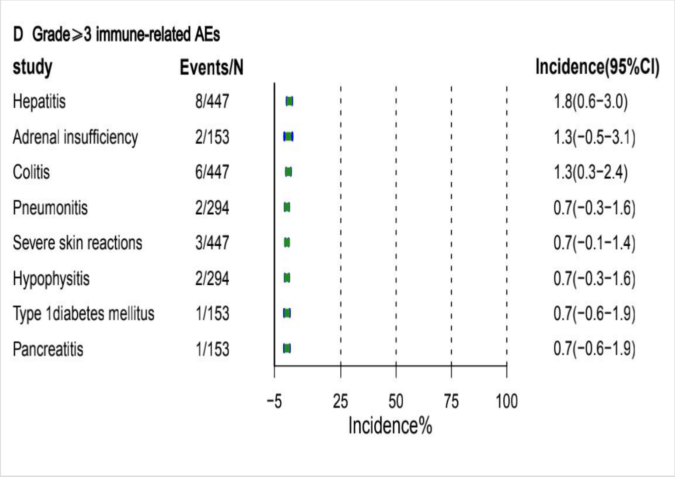

Supplementary Fig 8. Grade≥3 of PD-1 inhibitor irAEs adverse events

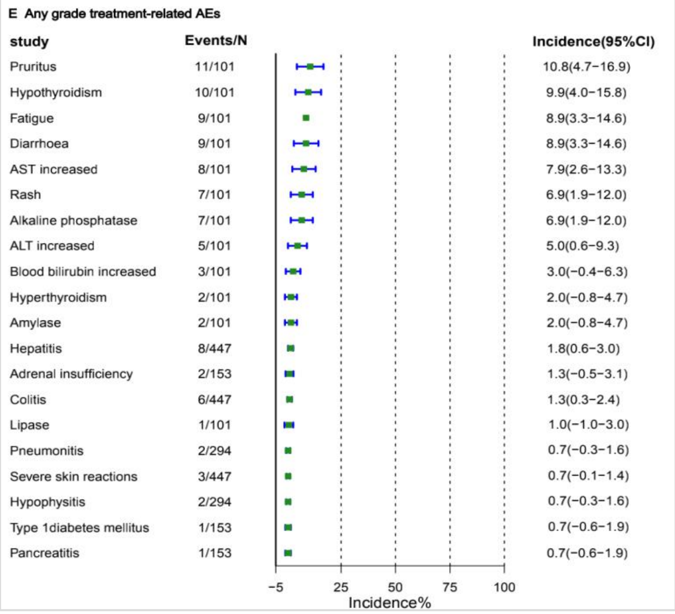

Supplementary Fig 9. Any grade of PD-L1 inhibitor trAEs adverse events

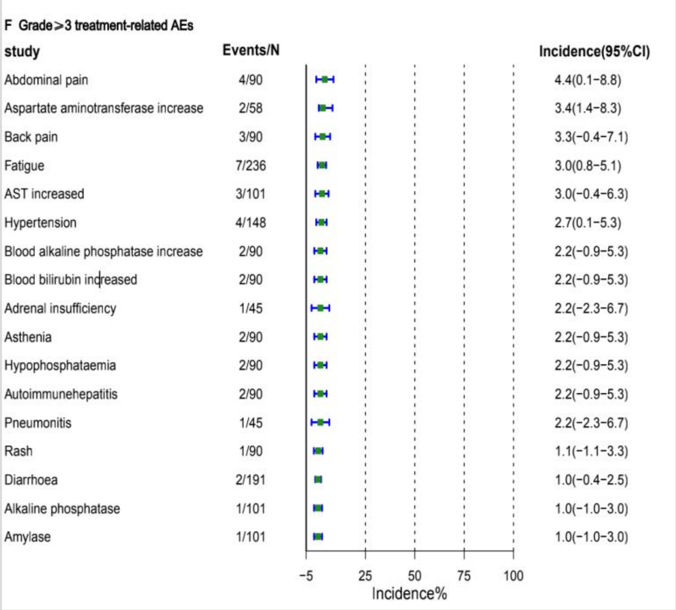

Supplementary Fig 10. Grade≥3 of PD-L1 inhibitor trAEs adverse events

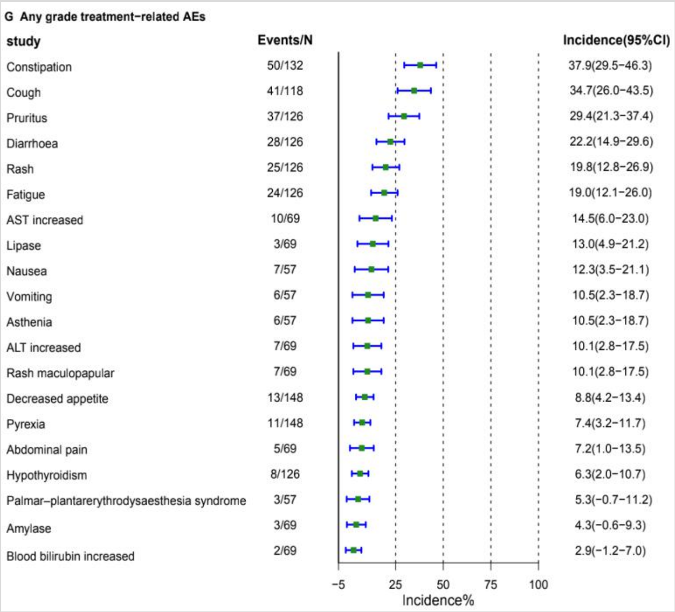

Supplementary Fig 11. Any grade of CTLA-4 inhibitor trAEs adverse events

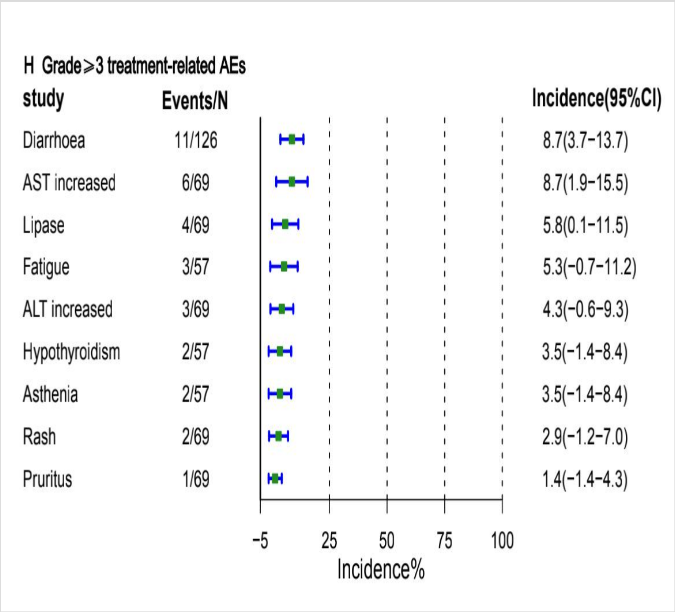

Supplementary Fig 12. Grade≥3 of CTLA-4 inhibitor trAEs adverse events

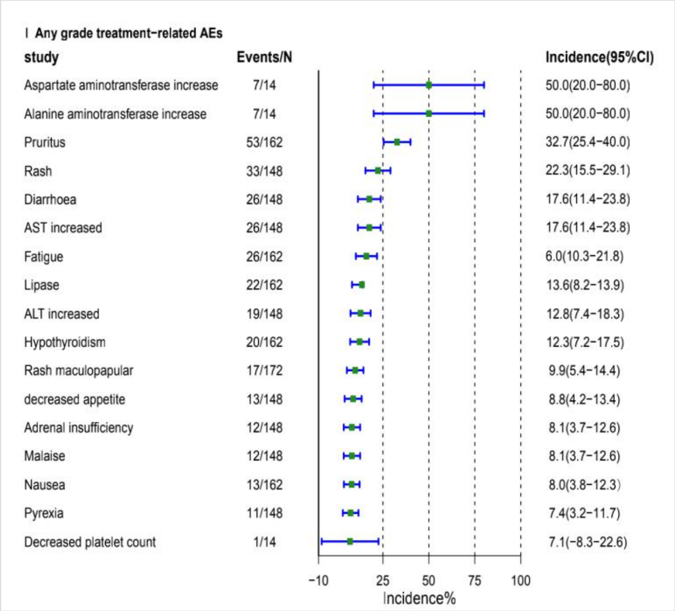

Supplementary Fig 13. Any grade of PD-1+CTLA-4 inhibitor trAEs adverse events

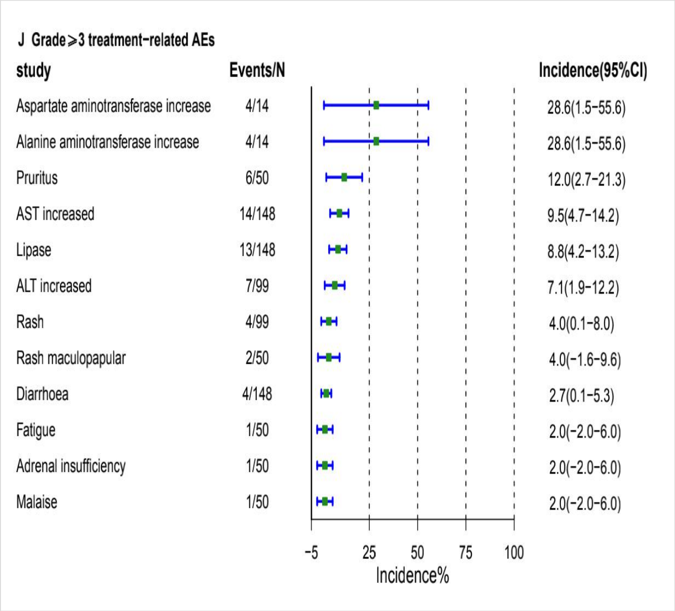

Supplementary Fig 14. Grade≥3 of PD-1+CTLA-4 inhibitor trAEs adverse events

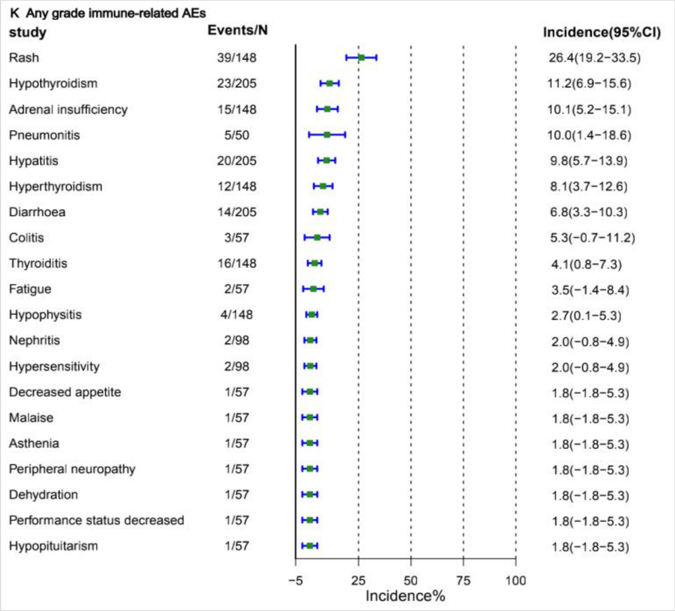

Supplementary Fig 15. Any grade of PD-1+CTLA-4 inhibitor irAEs adverse events

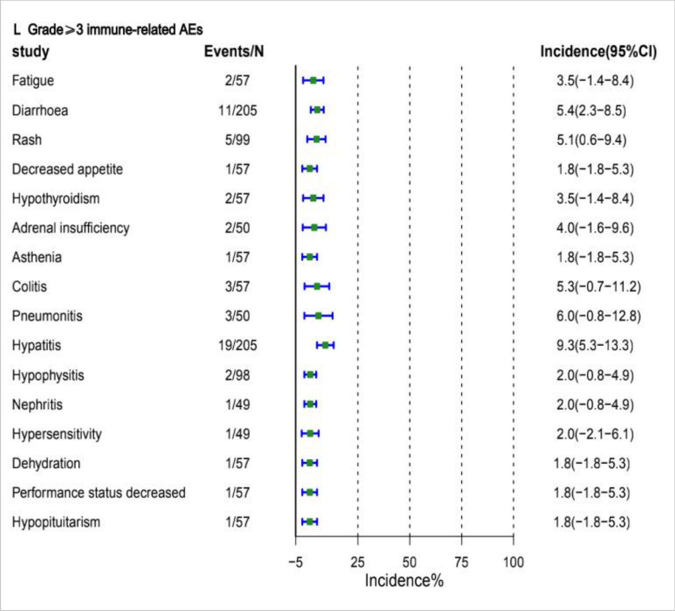

Supplementary Fig 16. Grade≥3 of PD-1+CTLA-4 inhibitor irAEs adverse events

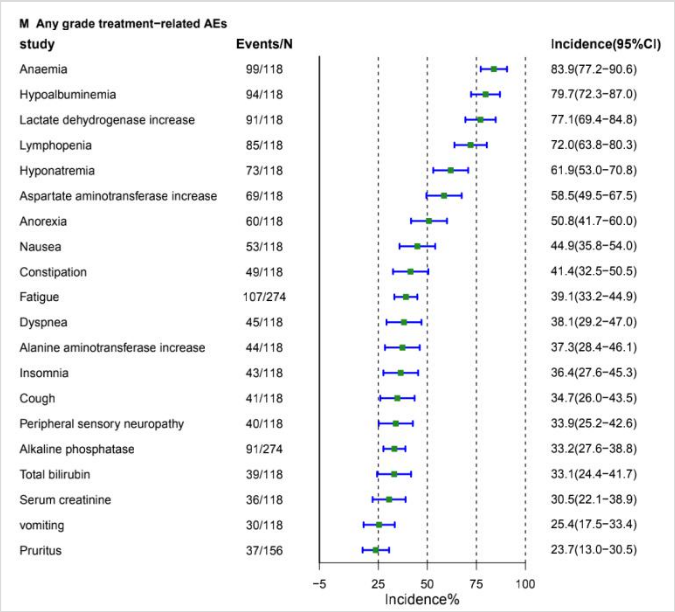

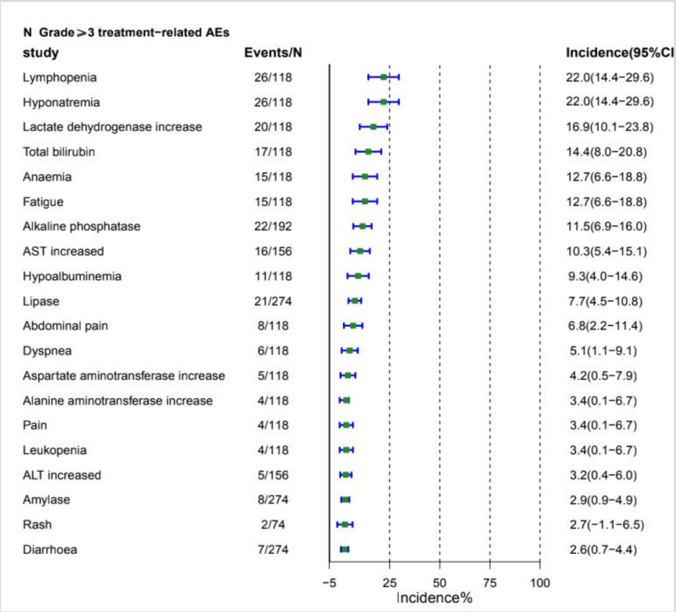

Supplementary Fig 17. Any grade of PD-L1+CTLA-4 inhibitor trAEs adverse events

Supplementary Fig 18. Grade≥3 of PD-L1+CTLA-4 inhibitor trAEs adverse events

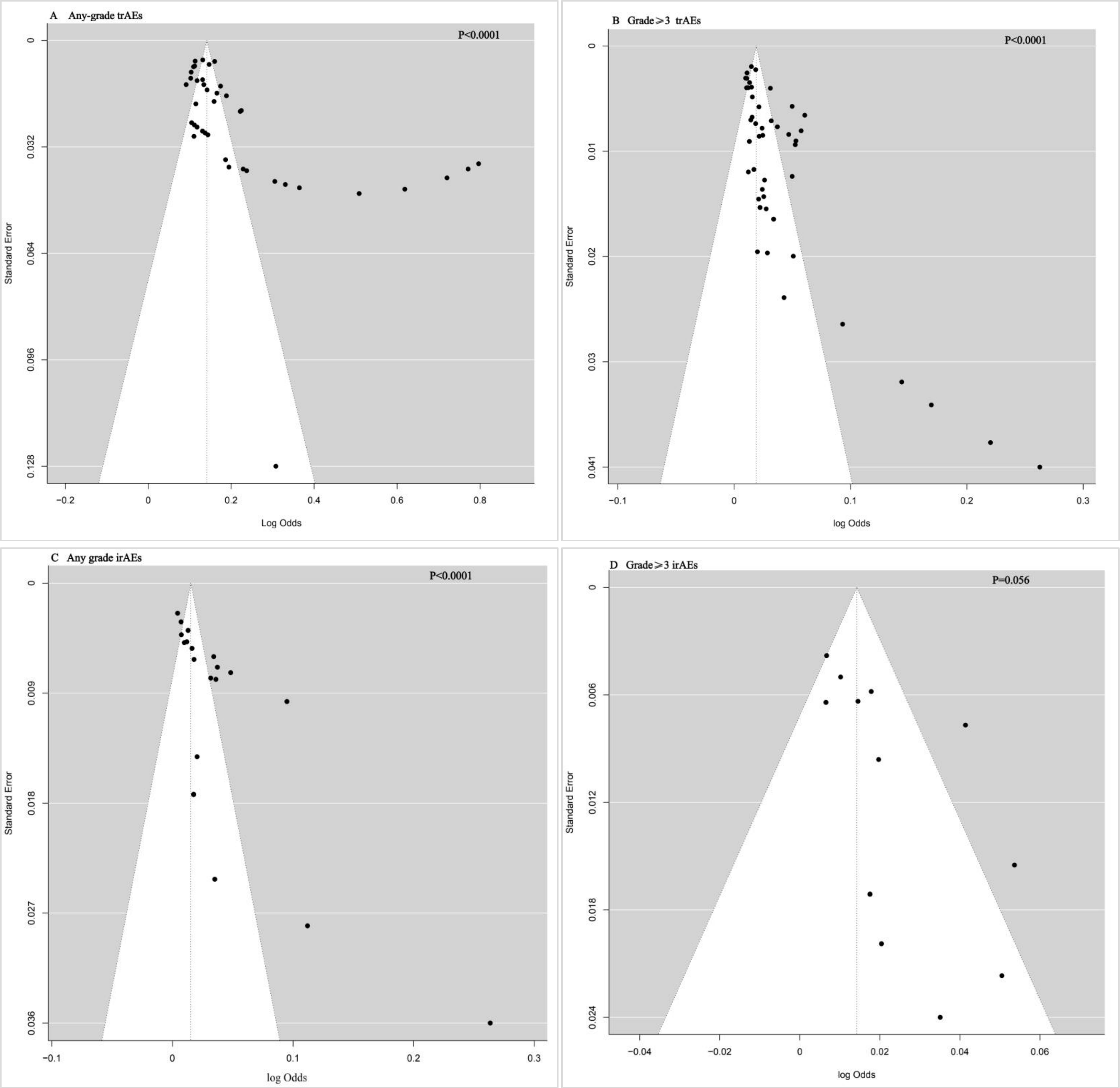

Supplementary Figure 19. Funnel plot of adverse event profile
